# Proteomic Analysis and *In Vivo* Studies Reveal the Potential Gastroprotective Effects of CHCl_3_ and Aqueous Extracts of *Ficus palmata*

**DOI:** 10.1155/2021/6613140

**Published:** 2021-05-30

**Authors:** Sameen Fatima Ansari, Arif-ullah Khan, Neelum Gul Qazi, Fawad Ali Shah, Komal Naeem

**Affiliations:** Riphah Institute of Pharmaceutical Sciences, Riphah International University, Islamabad, Pakistan

## Abstract

*Ficus palmata* is rich in several phytochemicals such as chromone, isoflavones, terpenes, lignans, coumarins, glycosides, and furanocoumarins and have been traditionally used for the management of different gastrointestinal disorders. This research reveals the effects of *Ficus palmata* fruit extracts—*Ficus palmata* chloroform (Fp.CHCl_3_) and *Ficus palmata* aqueous (Fp.Aq)—on gut activity through *in vivo* and *in vitro* analyses. Antidiarrheal and enteropooling assays were analyzed with castor oil-induced diarrhea and intestinal fluid accumulation. Jejunum tissues of rabbits were isolated (antispasmodic) for *in vitro* experiments. Antimotility was carried out by charcoal meal for determining transient time, and ethanol-induced ulcer assay was used to measure the ulceration of stomach; molecular pathways were assessed through proteomic approach. Fp.CHCl_3_ and Fp.Aq extracts attributed dose-dependently protection against diarrhea, and intestinal fluid secretions were inhibited dose dependently. Extracts of Fp.CHCl_3_ and Fp.Aq produced reduction in spontaneous and K^+^ (at 80 Mm)-induced contractions in isolated jejunum tissues, along with the decreased length covered by charcoal in charcoal meal transient time activity. The extract exhibited gastroprotective outcome in rats and reduced tumor necrotic factor (TNF-*α*) levels and IL-18, measured by proteomic approach. Morphological studies' results showed that ethanol induced significant gastritis, apoptosis, swelling of mucosa, and hydropic degeneration leading to cellular degeneration and necrosis, observed through staining techniques. Furthermore, ethanol activated the inflammation pathway in all gastric zones by elevating the levels of cyclooxygenase-2, TNF-*α*, and nuclear factor kappa light-chain enhancer of activated B-cells. Overall results expressed the antidiarrheal, antispasmodic, enteropooling, antimotility, and antiulcer activities of *Ficus palmata* fruit extract.

## 1. Introduction

Health care providers are highlighting the high occurrence of problems associated with the digestive system in Asian population, which plays a leading factor in comorbidities. In spite of recent great achievements and discoveries in medical science, there has been no effective treatment yet discovered with sufficient medical data supporting its efficacy for the cure of gastrointestinal disorders. The treatments available for such diseases are providing relief for short time but are also causing a number of other side effects, thus causing more harm than benefits. However, standard medical therapies involving plant extracts are economical and safe and are also providing relief from ailments for a longer period of time [1]. Herbal extracts and products are a part of a large number of modern medicines in fact; products extracted from herbal sources constitute 61% of the total globally launched drugs.

Plant extracts assessed based on their *in vivo* activity lead to many new therapies. Molecules showing promising biological activity are further incorporated in suitable dosage forms, and their dose and dosing regimen are decided in order to achieve required therapeutic effects. Edible fruits, provided as bioingredients and also separately as active ingredients, have provided cure for chronic gastrointestinal ailments. Treatment of gastrointestinal ailments using edible fruits has also been supported by various studies [2].

*Ficus palmata* Forsk. is one among the 800 species constituting the family Moraceae [3], which is normally known as “FegraFig” and “Injeer” in local term. This species is autochthonous of northern areas of Pakistan and the Himalayan region, which is known as wild Himalayan fig. Its family Moraceae includes mostly tall trees, shrubs and disconnected herbs mostly having milky juice [4]. A number of folk medicines showing tonic, anti-inflammatory and anti-tumor properties are extracted from members of this specie [5]. *Ficus palmata* extracts are reported to provide cure for ailments including tonsillitis, bronchitis, influenza, whooping cough, enteritis, epilepsy, jaundice, bacillary dysentery, toothache and bruises. *Ficus palmata* has exhibit antioxidant activity but also shows other pharmacological properties like nephroprotective, hepatoprotective and anticoagulant activities reported previously [6]. Different classes of compounds have been isolated e.g., chromone, isoflavones, terpenes, lignans, coumarins, glycosides, furanocoumarin and sterols from genus *Ficus palmata* after its extensive chemical analysis. 6 compounds including vanillic acid, germanicol acetate, psoralene, bergapten, psoralenoside, and flavone glycoside rutin have been isolated from the aerial parts of *Ficus palmata*.

Previously mentioned therapeutic uses of this genus have been validated by using folklore studies as baseline data [7, 8]. Furthermore, antisecretary, antimotility, antidiarrheal, antispasmodic, and antiulcer properties are being reported in this study. Ulcer is characterized as an inflammatory disorder, so here we hypothesize that the plant extract by inhibiting NF*κ*B-dependent proinflammatory cytokines can cure gastric ulcer. The current research aims to evaluate the possible effect of *Ficus palmata* in gastrointestinal activities including antidiarrheal, antimotility, enteropooling, antispasmodic, and antiulcer. Furthermore, in order to explore the underlying molecular mechanisms, molecular techniques including ELISA and IHC were employed to analyze the levels of inflammatory markers [9].

## 2. Materials and Methods

### 2.1. Materials

*Ficus palmata* fruits weighing 3 kg were purchased from marketplace in February 2018 and verified by Dr. Mushtaq Ahmad, a taxonomist at Quaid-i-Azam University, Islamabad (Department of Plant Sciences). The specimen sample of the particular species was submitted at the herbarium to obtain voucher specimen no. ISL-B-24. Further processing of fruits involved shade drying, crushing, and maceration in 500 mL chloroform ×4. Filtration of the whole extract was carried out by employing standard protocols via filter paper (Whatman No.1) followed by evaporation through a rotary evaporator, Tokyo Rikakikai Type A 1000S, at a lowered pressure and a temperature up to 50°C for generating dry extract in a yield of 20 g. After complete elimination of chloroform, sequential extraction of the leftover residue was carried out by suspending in a mixture of methanol and water (4 L, 20 : 80) via the above-mentioned technique. A 90 g yield of the extract was obtained by evaporating the solvent under a reduced pressure [9].

### 2.2. Drugs

Atropine, potassium chloride, acetylcholine, verapamil hydrochloride, activated charcoal powder, ethanol, methanol, papaverine, omeprazole, and loperamide hydrochloride were obtained from Sigma-Aldrich (Co, St Louis, MO, USA). Castor oil was purchased from KCL Pharma (Karachi, Pakistan).

### 2.3. Animals

Experimentations were carried out in accordance with the guidelines of the Riphah Institute of Pharmaceutical Sciences (Research and Ethics Committee; Ref. #REC/RIPS/2018/021) and the “Principles of Laboratory Animal Care.” Sprague-Dawley rats (190–270 g), rabbits (1.5–2.0 kg), and BALB/c mice (22–25 g) were used. All experimental animals were housed in the Riphah Institute of Pharmaceutical Sciences (RIPS), Islamabad, under optimum environmental conditions. All animals were placed in plastic cages at an optimum temperature (20–25°C) and 12-hour light/dark cycle. All animals received standard feed and tap water *ad libitum*. Each animal was subjected to a 24-hour fasting period before each experiment. Health status of animals was monitored twice daily during housing.

### 2.4. Phytochemical Tests

Qualitative phytochemical tests were performed on *Ficus palmata* extracts—chloroform (Fp.CHCl_3_) and aqueous (Fp.Aq)—for the presence of significant metabolites including tannins, saponins, glycosides, flavonoids, anthraquinones, proteins, alkaloids, and steroids as per the standard protocol with slight modifications [10].

### 2.5. Antidiarrheal Activity (Castor Oil-Induced Diarrhea)

Antidiarrheal activity was performed with slight modifications as previously described [11]. BALB/c mice were randomly assigned to five groups for Fp.CHCl_3_ and Fp.Aq and fasted for about 24 hours (08 : 00–08 : 00), before initiation of the experiment. Animals were placed in separate cages, lining the floors using absorbent paper. Negative control group animals received saline (10 mL/kg), while the positive control group received loperamide hydrochloride (2 mg/kg), and remaining groups received extract doses 50, 100, and 300 mg/kg. Castor oil (10 mL/kg p.o.) was administered to the mice after 1 hour following pretreatment. To analyze onset of diarrhea, the presence and absence of diarrheal dropping, animals were continuously monitored for about 4 hours. Results were evaluated according to Researcher Rehman [12] using the Chi-square (*X*^2^) test.

### 2.6. Measurement of Enteropooling Activity

Measurement of enteropooling assay was carried out by employing the previously described method [11]. Mice were randomly assigned into five groups with five mice in each and fasted for 24 hours (08 : 00–08 : 00). Group I received normal saline (10 mL/kg), and group II–V were administered castor oil p.o. (10 mL/kg), respectively. After 60 mins following pretreatment with castor oil p.o. (10 mL/kg), Fp.CHCl_3_ (50, 100, and 300 mg/kg, i.p.) was administered to groups II, III, and IV, respectively, while the standard group V received atropine (10 mg/kg). Similar dosing schedule was followed for Fp.Aq extract. After 30 mins, intestines of all the test animals were extracted and weighed following cervical dislocation. Data obtained were articulated as Pi/Pm × 1000 (Pi denotes weight in grams of intestine, and Pm denotes weight in grams of the animal) using GraphPad Prism and one-way analysis of variance (ANOVA) followed by *post hoc* Tukey's test.

### 2.7. Isolated Tissue Preparation

Rabbits were subjected to 24-hour fasting (08 : 00-08 : 00) before experimentation with free availability to water. Following cervical dislocation and jejunum extraction of rabbits, approximately 2 cm of the rabbit jejunum was immersed in the tissue organ bath filled with Tyrode's solution to simulate intestinal environment for 30 mins, along with continuous supply of oxygen (95% O_2_) and carbon dioxide (5% CO_2_) under controlled temperature prior to drug administration. ACh (0.3 *μ*M) was used to stabilize tissue, and response recording was accomplished through a force displacement transducer (model FT-03) joined with a bridge amplifier and power Lab 4/25 data acquisition system coupled with a computer running on Lab-Chart 6 software (AD Instrument, Sydney Australia) for recording the gastrointestinal motility effects of the plant extracts [13] and calculating % change in contractions of the jejunum [13]. GraphPad Prism was employed to analyze the data by applying sigmoidal dose-response (variable slope) following nonlinear regression curve.

### 2.8. Charcoal Meal Transit Time

Charcoal meal transit time was determined by the method described previously [9]. Rats were fasted for about 24 hours with free availability to water. Treatment groups were administered 50, 100, and 300 mg/kg (p.o.) of Fp.CHCl_3_ and Fp.Aq, respectively, and the standard group was given 0.1 mg/kg of atropine sulfate (i.p.), whereas 10 mL/kg of normal saline (p.o.) was administered to the negative control group. After 1 hour, charcoal meal was ingested to all groups. After 30 minutes following dose administration, all animals were subjected to cervical dislocation to extract the small intestine for the measurement of distance traveled by charcoal meal (5% activated charcoal suspension in distilled water) in the small intestine, as expressed by the following equation [11, 14]:

(1) $$\begin{array}{c} \text{peristaltic index }\left(PI\right)\%=\frac{\text{distance covered by charcoal meal}}{\text{total length of intestine}}\times \;100. \end{array}$$

The peristaltic index was then used for measuring % inhibition.

(2) $$\begin{array}{c} \%\text{ inhibition}=\frac{\left(PI{}_{C}-PI{}_{T}\right)}{PI{}_{C}}\times 100, \end{array}$$

where *PIC* denotes the peristaltic index of the control group, and *PIT* represents the peristaltic index of the test group.

### 2.9. Ethanol-Induced Ulcer Assay

Sprague-Dawley rats weighing 190–280 g were allocated into five random groups of either gender and fasted for 24 hours (09 : 00–09 : 00). Group 1 received 10 mL/kg normal saline; group II received standard drug omeprazole 20 mg/kg (p.o.); and groups III, IV, and V received 50, 100, and 300 mg/kg (p.o.) of Fp.CHCl3 and Fp.Aq extracts, respectively. After 60 minutes following treatment, all animals were administered 100% ethanol of 1 mL/100 g (p.o.) for induction of gastric ulcer. All animals were subjected to cervical dislocation to extract the stomach for the estimation of lesion index via measurement of the greater curvature of each lesion in millimeters. Scoring comprised measuring of the surface area of each lesion. Ulcer index was expressed as mean US (ulcer score) for each lesion (0: no ulcer; 1: US ≤ 0.5 mm^2^; 2: 0.5 < US ≤ 2.5 mm^2^; 3: 2.5 mm^2^ < US ≤ 5 mm^2^; 4: 5 mm^2^ < US ≤ 10 mm^2^; 5: 10 mm^2^ < US ≤ 15 mm^2^; 6: 15 mm^2^ < US ≤ 20 mm^2^; 7: 20 mm^2^ < US ≤ 25 mm^2^; 8: 25 mm^2^ < US ≤ 30 mm^2^; 9: 30 mm^2^ < US ≤ 35 mm^2^; and 10: US > 35 mm^2^) [15]. Data obtained for each stomach ulcer were expressed as ulcer index (UI), which is the sum of lengths of all sores in millimeters. The assessment of gastric protection was expressed as percent inhibition estimated using the following formula:

(3) $$\begin{array}{c} \%\text{ inhibition}=\;\frac{\left(USc-USt\right)}{USc\;}\;\times \;100, \end{array}$$

where *USc* denotes the ulcer surface area of control, and *USt* denotes the ulcer surface area of the test drug group.

### 2.10. Proteomic Analysis Using ELISA

TNF-*α*, PGE-2, and IL-18 expressions were measured using the Rat TNF-*α*, PGE-2, and IL-18 ELISA kit according to the manufacturer's instructions (Elabscience). The tissues were homogenized at 1500 rpm by means of Silent Crusher M (Heidolph), and the supernatant was recovered following centrifugation (1350 ×g, 1 hour). Concentrations of PGE-2, IL-18, and TNF-*α* were measured using the ELISA microplate reader [16]. Results were represented in picograms per milliliter (pg/mL).

### 2.11. Tissue Collection for Morphology Analysis, Histopathology, and Immunohistochemistry

For morphological examination, 5 rats were included in each experimental group. Stomach tissues were immersed in 4% formalin followed by embedding in paraffin till further analysis. Later on, tissue sectioning was done via a rotary microtome at 5 *μ*m followed by hematoxylin and eosin staining (H&E). The stained gastric tissues were then analyzed under an optical microscope, and images were taken by the same procedure as described previously [17]. Immunohistochemical staining was performed as mentioned by Shah et al. [17].

Deparaffinization of tissue sections coated on the slides was carried out by using three different solutions of absolute xylenes followed by rehydration with ethanol in descending order of concentrations (100%–70%). Slides were then stored for 10 mins in 0.01 M phosphate-buffered saline (PBS) after rinsing with distilled water. After the antigen retrieval step, the slides were left for incubation for overnight with primary antibody, by next day treatment with appropriate biotinylated secondary antibodies for about 2 hours, and successively with avidin-biotin complex (ABC) reagents (Standard Vectastain ABC Elite Kit; Vector Laboratories, Burlingame, CA, United States) for approximately 1 hour at optimum room temperature. The sections were washed with PBS and stained in 3,3′-diaminobenzidine (DAB) solution as a chromogen; they were then washed with distilled water, dehydrated in graded concentrations of ethanol (70, 95, and 100%), then fixed in xylene, and cover-slipped by a mounting media and left for air drying. A light microscope (Olympus, Japan) was used for result analysis, coupled with a high-quality digital photo microscopy system. TIF images of immunohistochemistry slides (five images per slide) were taken. Further quantification of hyperactivated COX-2, TNF-*α*, and p-NF-*κ*B (Santa Cruz Biotechnology) antibodies was accomplished through ImageJ software.

### 2.12. Acute Toxicity

The current study explores the *in vivo* effect of extract by employing an acute toxicity model. Mice were allocated into three groups (*n* = 5). The test was conducted by using escalating doses of Fp extract, i.e., 3 and 5 g/kg given in 10 mL/kg volume. Saline (10 mL/kg, p.o) served as a negative control. Each animal was individually monitored for about 4 hours to detect any behavioral changes, and determination of mortality rate was after continuous observation for 24 hours following dose completion [18].

## 3. Results

### 3.1. Phytochemistry of Extracts

Fp.CHCl_3_ and Fp.Aq extracts indicate the presence of glycosides, phenols, flavonoids, alkaloids, coumarins, furanocoumarins, saponins, triterpenes, carbohydrates, lignin, and unsaturated sterols.

### 3.2. Determination of Fp.CHCl_3_ and Fp.Aq on Castor Oil-Induced Diarrhea

Fp.CHCl_3_ and Fp.Aq displayed a protective effect in a dose-dependent manner against castor oil-induced diarrhea in mice, while no protection was observed in the control group (saline treated). Fp.CHCl_3_ exhibited a marked response of 60% protection from diarrhea at 50 mg/kg, 80% at 100 mg/kg, and 100% protection at 300 mg/kg. On the other side, Fp.Aq extract showed 40%, 60%, and 80% (*p* < 0.05 vs. saline group) inhibition at doses of 50, 100, and 300 mg/kg, respectively. Loperamide at a dose of 2 mg/kg possessed antidiarrheal effect and 100% protection from diarrhea (*p* < 0.01 vs. saline group) in the positive control group as shown in Table 1.

### 3.3. Determination of Fp.CHCl_3_ and Fp.Aq on Enteropooling Assay

**Figure 1:**
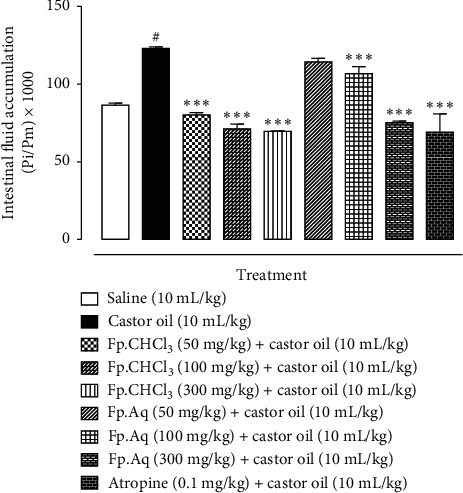
Inhibitory effect of *Ficus palmata* extracts: chloroform (Fp.CHCl_3_), aqueous (Fp.Aq), and atropine on castor oil-induced fluid accumulation in mice. Results are expressed as mean ± SEM, *n* = 5. Antisecretory effect is expressed as Pi/Pm × 1000 (g), where Pi is the weight of the small intestine and Pm is the weight of the mouse. ^#^*p* < 0.001 vs. saline group and ^*∗∗∗*^*p* < 0.001 vs. castor oil group, analyzed by one-way analysis of variance with post hoc Tukey's test.

For determining, enteropooling assay castor oil is used; Fp.CHCl_3_ and Fp.Aq exhibited antisecretory effect in a dose-dependent manner, i.e., 50–300 mg/kg. With reference to the saline group, intestinal fluid accumulation was 86.5 ± 0.93 (mean ± SEM, *n* = 5); however, the values were higher for the castor oil-treated group, i.e., 123 ± 0.9. Fp.CHCl_3_ at an increasing level of doses, i.e.,50, 100, and 300 mg/kg, deducted the accumulation of castor oil-induced fluid to 80.18 ± 1.5 (*p* < 0.001 vs. castor oil group), 71.25 ± 2.95 (*p* < 0.001 vs. castor oil group), and 70.588 ± 0.316 (*p* < 0.001 vs. castor oil group), respectively, whereas the activity of Fp.Aq at the doses of 50, 100, and 300 mg/kg reduced the castor oil-induced fluid accumulation to 114.32 ± 2.11 (*p* < 0.001 vs. castor oil group), 106.6351 ± 2.09 (*p* < 0.001 vs. castor oil group), and 75.114 ± 0.42 (*p* < 0.001 vs. castor oil group), respectively. Atropine at the dose of 10 mg/kg decreased the intestinal fluid accumulation to 75.114 ± 0.42 (*p* < 0.001 vs. castor oil group) as shown in Figure 1.

### 3.4. Determination of Fp.CHCl_3_ and Fp.Aq on Spontaneous and K^+^-Induced Contractions

**Figure 2:**
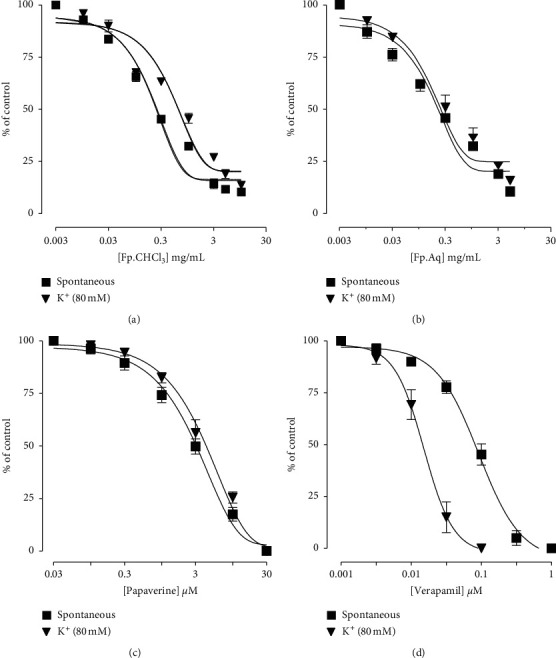
Dose-dependent inhibitory effect on spontaneous and K^+^ (80 mM)-induced contractions of *Ficus palmata* extracts: (a) chloroform (Fp.CHCl_3_), (b) aqueous (Fp.Aq), (c) papaverine, and (d) verapamil in isolated tissue preparations. Results expressed as mean ± SEM, *n* = 3–5.

The comparative inhibitory effect of the plant extracts shown by papaverine and verapamil is in oppose to spontaneous and K^+^ (80 mM)-induced contractions. Fp.CHCl_3_ was observed as effective in oppose to spontaneous and K^+^ (80 mM)-induced movements with the EC_50_ values of 0.2045 mg/mL (0.1557 to 0.2687, *n* = 3-4) and 1.085 mg/mL (0.2070 to 5.686, *n* = 3-4), respectively, as shown in Figure 2(a). Similarly, Fp.Aq showed spontaneous and K^+^ (80 mM)-induced contractions with the EC_50_ values of 0.4811 mg/mL (0.02345 to 9.870, *n* = 4) and 0.2377 mg/mL (0.07131 to 0.7922, *n* = 4), respectively, as shown in Figure 2(b). Papaverine also showed the similar pattern of nonspecific inhibitory response (Figure 2(c)) with respective EC_50_ values of 0.4 *μ*M (0.2-0.8, *n* = 4) in spontaneous and 0.6 (0.3-1.3, *n* = 4) in high K^+^-induced contractions, whereas verapamil was found more potent against K^+^ (80 mM)-induced contractions with an EC_50_ value of 0.04 *μ*M (0.03-0.06, *n* = 4) compared with spontaneous contractions (0.12 *μ*M (0.10–0.20, *n* = 3)) as shown in Figure 2(d).

### 3.5. Effect of Fp.CHCl_3_ and Fp.Aq on Charcoal Meal Transit Time

Fp.CHCl_3_ delayed traveling of charcoal meal through the small intestine in a dose-dependent manner. In the saline group, the distance traveled was 92.6%. Fp.CHCl_3_ at 50, 100, and 300 mg/kg doses showed % inhibition in charcoal meal transit by 12.24, 14.57, and 18.97%, respectively; whereas Fp.Aq showed % inhibition of 19.98%, 25.10%, and 32.39% at 50, 100, and 300 mg/kg doses (*p* < 0.001 vs. saline group). Atropine used as a standard drug (0.1 mg/kg, i.p.) exhibited an inhibitory effect of 82.31% (Table 2).

### 3.6. Effect of Fp.CHCl_3_ and Fp.Aq on Ulcerative Stomach

**Figure 3:**
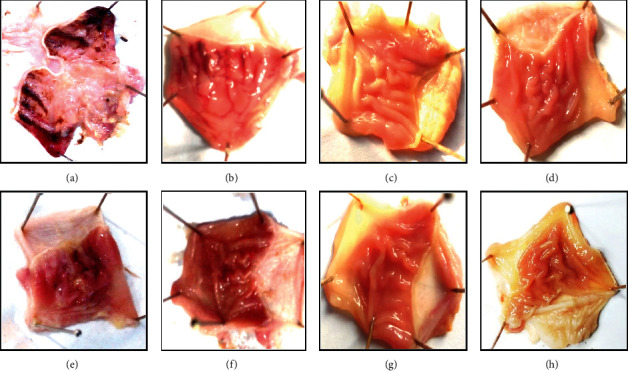
Gross appearance of gastric mucosa in rats: (A) pretreated with saline, 10 mL/kg (ulcer control). Severe injuries are seen, as ethanol (1 mL/100 g) produced excessive hemorrhagic necrosis of gastric mucosa pretreated with *Ficus palmata* extracts: (B, C, D) chloroform (Fp.CHCl3); (E, F, G) aqueous (Fp.Aq) at doses of 50, 100, and 300 mg/kg, respectively; and (H) pretreated with omeprazole (20 mg/kg). The injuries reduce with the increase of Fp.CHCl_3_ and Fp.Aq doses and omeprazole compared with ulcer control. At 300 mg/kg, Fp.CHCl_3_ and Fp.Aq showed the most efficacious gastroprotective action.

Fp.CHCl_3_ relieved the ulcerative stomach at 50–300 mg/kg in a gradually increasing dose-effective manner to antiulcer effect. Fp.CHCl_3_ at 50, 100, and 300 mg/kg caused 20.40, 55.10, and 75.51% (*p* < 0.001 vs. saline group) inhibition, respectively. However, omeprazole, a standard drug at 20 mg/kg, exhibited 86% inhibitory effect. Fp.Aq showed a positive increase in percent inhibition of ulcerative stomach of rats by healing wound at a dose of 300 mg/kg, and % inhibition was 35.41%, 54.16%, and 75% observed at 50, 100, and 300 mg/kg (Table 3). The ulcer surface area could be visually examined (Figure 3).

### 3.7. Effect of Fp.CHCl_3_ and Fp.Aq on TNF-*α*, IL-18, and PGE-2 Expressions in Ulcerative Stomach of Rats

**Figure 4:**
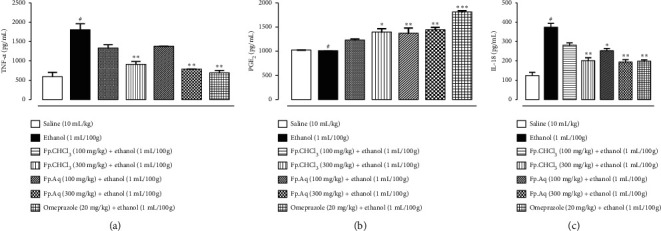
Effect of *Ficus palmata* extracts: chloroform (Fp.CHCl_3_) and aqueous (Fp.Aq) in comparison with saline, ethanol, and omeprazole groups on TNF-*α*, PGE-2, and IL-18 in the ethanol-induced ulcer model. The gastric tissue was homogenized as described in the Methods section, and TNF-*α* levels were measured by ELISA assay. ^*∗*^*p* < 0.05, ^*∗∗*^*p* < 0.01, and ^*∗∗∗*^*p* < 0.001, analyzed by one-way ANOVA followed by Tukey's *post hoc* test.

For examination of TNF-*α*, IL-18, and PGE-2 in animal tissue, ELISA was performed in gastric tissues. The results significantly decreased in level of the treated group vs. disease group (^*∗∗∗*^*p* < 0.001 vs. ethanol group) was analyzed by one-way analysis of variance (ANOVA) followed by *post hoc* Tukey's test. The higher doses of Fp.CHCl_3_ produced less pronounce effect compared with Fp.Aq. The graphical representation of designated antibody levels is shown in Figure 4.

### 3.8. Tissue Analysis Using Histopathology and Immunohistochemistry (IHC)

**Figure 5:**
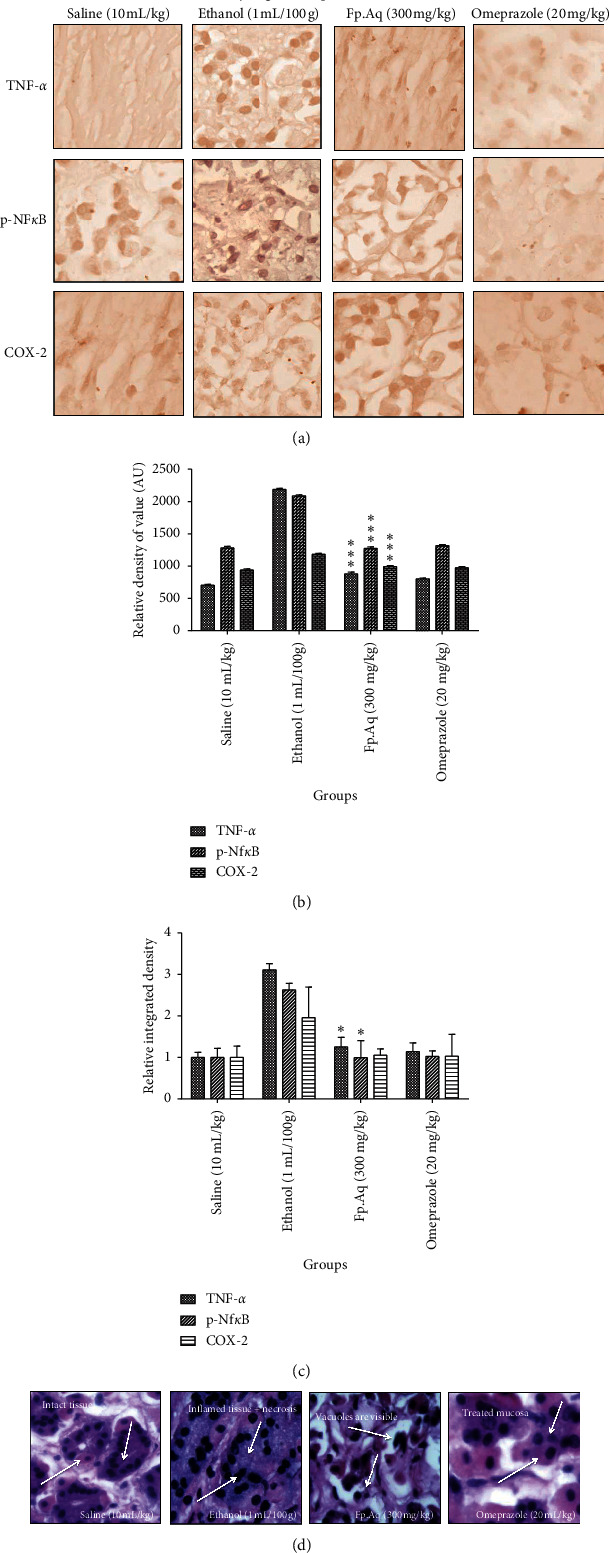
Effects of (Fp.Aq) extract on ethanol-mediated stomach histopathologic changes. Stomach tissues (*n* = 5) from each experimental group were processed for histological evaluation at 1 h after ethanol challenge. (A) Histological changes of apoptotic markers (TNF-*α*, P-NF*κ*B, and COX-2) in the stomach, scale bar = 20 *μ*m, magnification 40X in the control group, ethanol group, Fp.Aq extract group, and standard omeprazole group. (B, C) Severity scores of stomach injury in different groups (*n* = 5) calculated via relative density of value (A.U.) and relative integrated density. Data were analyzed by two-way ANOVA followed by post hoc Tukey's test using GraphPad Prism software in mean ± SEM. ^*∗∗∗*^*p* < 0.001 vs. ethanol group and ^*∗*^*p* < 0.01 vs. ethanol group. (D) Histopathological examination of the saline group, ethanol group, test group, and omeprazole group.

For tissue analysis, IHC and H&E staining were performed to distinguish necrotic cells from healthy ones. Ethanol by generating biochemical events produced robust cellular changes in prone areas of gastric cells, with significant hypertrophic appearance in tissues revealed through immunohistochemistry analysis as shown in Figure 5. A significant increase in the expression of TNF-*α*, p-NF*κ*B, and COX-2 was noticed in the disease-induced group relative to the saline group, validated through ELISA protein assay in which significant elevations of these markers were seen. The effects were significantly reversed by pretreatment with Fp.Aq doses. Furthermore, results were analyzed on the basis of mucosal cell size, shape, staining, and vacuolation using ImageJ software. Measurements were drawn in GraphPad Prism to calculate the relative density of value (A.U.) and relative integrated density (mean ± SEM).

### 3.9. Acute Toxicity

The Fp.CHCl_3_ and Fp.Aq extracts did not produce any mortality, and no significant behavioral and pathological changes were observed at the dose of 5 g/kg.

## 4. Discussion

For ethnopharmacological utilization of *Ficus palmata* in gastrointestinal disorders, including gastritis, constipation, and diarrhea, plant extracts were evaluated for its antidiarrheal, antispasmodic, charcoal meal gastrointestinal motility, and antiulcer effects. For evaluation of its ethnomedicinal uses, *in vitro* and proteomic analyses were used for the exploration of possibly underlying mechanism(s).

In *Ficus palmata* extract, a variety of group of compounds are present: flavonoids, polyphenols, alkaloids, carbohydrates, saponins, and anthraquinones. Furthermore, polyphenol metabolites mainly target gut microbiota and are responsible for its functioning [19] through these phytochemical constituents. *Ficus palmata* may remain accountable for its curative action in diarrhea; still the underlying mechanism is not further explored, which will identify the precise nature of chemical constituents involved in alleged biological activities.

Loperamide, an established standard drug for its protection against diarrheal disorder, extracted in same manner, exhibited protective effect against diarrhea; furthermore, mechanism elucidation was carried out through isolated tissue preparation technique, interlinking gastric motility. Ricinoleic acid, an active metabolite of castor oil, is responsible for the accumulation of enteropooling along with laxative effect [11]. It alters water movement and electrolyte levels along with the generation of contractions in transverse and distal sections of the colon. Histamine, acetylcholine, prostaglandins, substance P, nitric oxide, 5-hydroxytrptamine, and cholecystokinins are key components that maintain the gastric motility that provides stimulatory effects in the gut and ultimately increase cytosolic Ca^2+^ along with the release of mediators that block the above-described pathways in accompanying with nonspecific inhibitory action, which ultimately relieves gut disorders. The extracts provide evidence of protective effect against castor oil-induced intestinal fluid secretion in mice [12]. Extracts contain a gut relaxant constituent, which mediates antidiarrheal and antisecretory effects.

In transient time activity, extracts produce suppression of the propulsion of charcoal marker in the small intestine just like the standard drug atropine sulphate, which is known for its prominent anticholinergic effect on the intestine. By diminishing GIT motility tone, it results in increased stay of substances in the intestine, which permits better water absorption. The finding claimed that the plant extract has significant effects on the peristaltic movement of intestine, which indicates its antimotility effects.

Ulcers are sores produced by the disturbed production of enzymes and acids, which results in rupturing of mucosa and exposure of gastric walls. Various protective and aggressive factors are responsible to maintain balance in the gastrointestinal tract [20]. Through ethanol-induced assay, the beneficial effects of *Ficus palmata* extracts were explored. Generation of free radicals and superoxide anion, mucus exhaustion and damage, increased levels of oxidative enzyme, and stimulation of NO pathway are key factors that tend to release inflammatory mediators such as TNF-*α*, PGE-2, p-NF*κ*B, and COX-2 quantified by proteomics. The antiulcer activity of extract may refer its mechanism similar to CCB, as Ca^+2^ is known for such effects as explored earlier [21]. *Ficus palmata* has been reported for its antioxidant and nitric oxide free radical scavenging activities, so the antiulcer effect is due to its antioxidant property [22]. Fp.Aq in comparison with chloroform fraction has more potency to cure ulcerogenic stomach, so Fp.Aq treatment in animals was used for further analysis of IHC and H&E staining [16]. Moreover, no mortality was observed in the acute toxicity model at doses of 3 and 5 g/kg [20].

## 5. Conclusions

*Ficus palmata* has been traditionally used for treatment of diseases for a longer time. This study proves its beneficial effects in treating gastric disorders by attenuating oxidative stress and inflammatory pathways, ultimately account for gastroprotective effects against different diseases.

## Figures and Tables

**Table 1:** Effect of *Ficus palmata* extracts: chloroform (Fp.CHCl_3_), aqueous (Fp.Aq), and loperamide by using castor oil induced diarrhea in mice.

Treatment (mg/kg)	No. of mice (out of 5) with diarrhea	Protection (%)
Saline (10 mL/kg) + castor oil (10 mL/kg)	5/5	0
Fp.CHCl_3_ (50 mg/kg) + castor oil	2/5^*∗*^	60
Fp.CHCl_3_ (100 mg/kg) + castor oil	1/5^*∗*^	80
Fp.CHCl_3_ (300 mg/kg) + castor oil	0/5^*∗∗*^	100
Fp.Aq (50 mg/kg) + castor oil	3/5	40
Fp.Aq (100 mg/kg) + castor oil	2/5^*∗*^	60
Fp.Aq (300 mg/kg) + castor oil	1/5^*∗*^	80
Loperamide (10 mg/kg) + castor oil	0/5^*∗∗*^	100

^*∗*^ *p* < 0.05 and ^*∗∗*^*p* < 0.01 vs. saline + castor oil-treated group (Χ^2^-test).

**Table 2:** Effect of *Ficus palmata* extracts: chloroform (Fp.CHCl_3_), aqueous (Fp.Aq), and atropine on charcoal meal transit time in rats.

Doses (mg/kg, p.o.)	Mean length of intestine (cm)	Distance moved by charcoal (cm)	% Intestinal transient	% Inhibition
Saline (10 mL/kg)	92.6 ± 1.6	90 ± 1.3	97.1	-
Fp.CHCl_3_ (50 mg/kg)	79.8 ± 1.6	68 ± 1.7	85.21	12.24
Fp.CHCl_3_ (100 mg/kg)	79.8 ± 1.6	66.2 ± 1.0	82.95	14.57
Fp.CHCl_3_ (300 mg/kg)	78.8 ± 0.3	62 ± 2.0	78.68	18.97
Fp.Aq (50 mg/kg)	79.8 ± 0.6	62 ± 0.9	77.69	19.98
Fp.Aq (100 mg/kg)	77 ± 0.8	56 ± 0.8^∗∗^	72.72	25.10
Fp.Aq (300 mg/kg)	47 ± 1.3	71.6 ± 0.5^∗∗∗^	65.64	32.39
Atropine (0.1 mg/kg, i.p.)	90.8 ± 0.9	15.6 ± 0.6^∗∗∗^	17.18	82.31

^*∗∗∗*^ *p* < 0.001 vs. control (saline) group, one-way ANOVA followed by *post hoc* Tukey's test, *n* = 5.

**Table 3:** Protective effect of *Ficus palmata* extracts: chloroform (Fp.CHCl_3_), aqueous (Fp.Aq), and omeprazole against ethanol-induced gastric ulcers in rats.

Treatment	Ulcer index	% Inhibition
Saline 10 mL/kg + ethanol	10.0 ± 0.0	-
Fp.CHCl_3_ (50 mg/kg) + ethanol	7.8 ± 0.37^*∗*^	20.40
Fp.CHCl_3_(100 mg/kg) + ethanol	4.4 ± 0.24^*∗∗*^	55.10
Fp.CHCl_3_ (300 mg/kg) + ethanol	2.4 ± 0.24^*∗∗∗*^	75.51
Fp.Aq (50 mg/kg) + ethanol	6.2 ± 0.37^*∗*^	35.41
Fp.Aq (100 mg/kg) + ethanol	4.4 ± 0.24^*∗∗*^	54.16
Fp.Aq (300 mg/kg) + ethanol	2.4 ± 0.24^*∗∗∗*^	75
Omeprazole (20 mg/kg) + ethanol	1.4 ± 0.24^*∗∗∗*^	86

^*∗∗∗*^ *p* < 0.001 vs. control saline group, one-way analysis of variance followed by *post hoc* Tukey's test, *n* = 5.

## Data Availability

The data sets used and/or analyzed during the current study are available from the corresponding author on reasonable request.

## Abbreviations

Fp.CHCl_3_: *Ficus palmata* chloroform extract
Fp.Aq: *Ficus palmata* aqueous extract
K^+^: Potassium
kg: kilogram
mL: milliliter
p.o: Per oral
CO_2_: Carbon dioxide
O_2_: Oxygen
g: Gram
cm: Centimeter
ACh: Acetylcholine
USc: Ulcer surface area of control
USt: Ulcer surface area of test drug group.
